# Tumor, host and surgery related factors predisposing to cranial nerve deficits after surgical treatment of parapharyngeal space tumors

**DOI:** 10.1007/s00405-020-06261-8

**Published:** 2020-08-10

**Authors:** Anna Rzepakowska, Ewa Osuch-Wójcikiewicz, Michał Żurek, Aneta Durmaj, Kazimierz Niemczyk

**Affiliations:** 1 Department of Otorhinolaryngology Head and Neck Surgery, Medical University of Warsaw, ul. Banacha 1a, 02-097 Warsaw, Poland; 2 Students Scientific Research Group at the Department of Otorhinolaryngology Head and Neck Surgery, Medical University of Warsaw, Warsaw, Poland

**Keywords:** Parapharyngeal space, Surgery, Cranial nerve palsy

## Abstract

**Propose:**

Identification of relevant features acquired on preoperative evaluation of parapharyngeal space (PPS) tumors or related to the performed surgical approach that are predictive of the most important complication of surgical treatment of these tumors, cranial nerve palsy.

**Methods:**

This was a retrospective analysis of 68 patients with PPS tumors treated with surgical resection in a tertiary referral center from 2009 to 2019. The preoperative clinical symptoms, age, sex, tumor size, location, histopathological type, surgical approach, radical resection, intraoperative bleeding and the occurrence of complications were collected, evaluated and compared.

**Results:**

Cross-table and chi-square test results revealed that cranial nerve deficits were more common in neurogenic tumors than in other types, including malignant tumors (*χ*^2^ = 6.118, *p* = 0.013); the cervical approach was selected more often for neurogenic tumors (*χ*^2^ = 14.134, *p* < 0.001); neurogenic tumors were more frequently removed intracapsularly (*χ*^2^ = 6.424, *p* = 0.011); and neurogenic tumors were more likely to be located in the poststyloid area (*χ*^2^ = 17.464, *p* < 0.001). The two-sample *t* test revealed a significant correlation between age and the prevalence of cranial nerve complications (*t* = 2.242, *p* = 0.031). The mean age in the group of patients with cranial nerve palsy was 45.89 years, and that of the group without complications was 54.69 years. The results of logistic regression confirmed that the risk of nerve deficits was almost 8 times higher for neurogenic tumors (OR = 7.778, *p* = 0.01). None of the other analyzed variables related to tumor or surgery was significantly correlated with an increased risk of cranial nerve dysfunction.

**Conclusion:**

Surgical resection of tumors other than neurogenic tumors of the PPS reveals no significant risk for permanent neural dysfunction. Tumor size also had no significant effect on the risk of postoperative nerve palsy.

## Introduction

Parapharyngeal space (PPS) tumors are rare pathologies that comprise approximately 0.5% of all head and neck tumors [1, 2]. Due to the variety of structures surrounding and within the PPS, approximately 70 different histological types of parapharyngeal pathologies have been described so far [2]. Eighty percent of them are benign, and 20% are malignant. The most common lesions arise from the salivary glands, followed by neurogenic tumors [1–4].

Although PPS tumors are rare, they are still challenging both for diagnosis and therapy. They develop in deeply located anatomical spaces, and the majority of lesions have a slow progression and, therefore, are usually asymptomatic for a long time [5, 6]. Occult localization of this region prevents diagnosis on routine examination because the PPS is inaccessible both during palpation and with ultrasound [7]. Asymmetry of the lateral pharyngeal wall is the most commonly demonstrated symptom. Pain or paralysis of the cranial nerves may be indicators of malignancy [5]. However, PPS tumors are quite commonly detected by coincidence during screening for other reasons [8].

Computed tomography (CT) or magnetic resonance imaging (MRI) is crucial for the assessment of PPS tumors. They provide information about tumor size, location and relationship to the surrounding structures [6]. Tumor assignment to the pre- or poststyloid compartment facilitates a potential diagnosis. Lesions in the prestyloid space usually derive from the parotid gland, whereas those in the poststyloid space are usually of neurogenic origin [2, 6]. Preoperative imaging is also crucial for distinguishing between benign and malignant tumors. Radiological signs of malignancy include irregular tumor margins, infiltration in surrounding soft tissues, regional metastasis and evidence of enlarged, necrotic lymph nodes in the retropharyngeal and cervical areas [4]. The CT and MRI findings are important for making decisions about the appropriate surgical approach and complete resection with minimal morbidity [2, 6].

Surgery is the standard PPS tumor treatment. Information obtained on clinical evaluation and with imaging methods are valid for stratifying tumor histology and significantly influence the selection of surgical approach, extent of the resection, and the decision to perform tumor embolization prior to surgery as well as enable the prediction of possible complications and the planning of rehabilitation [2–4]. The three main approaches applied in the surgical treatment of parapharyngeal space pathologies are transcervical, transparotid–transcervical and transoral. The transmandibular procedure is currently less frequently used. The most preferred approaches are a transcervical route for tumors in the prestyloid compartment and a transparotid–transcervical route for tumors located in the poststyloid space [2, 4]. The transoral resection of PPS tumors should be restricted for selected small and benign lesions located in the prestyloid space due to the limited exposure and the lack of control of the major neck vessels and cranial nerves [9].

The challenge for surgery of PPS tumors results not only from poor access to that area, but also from the vascular and neural structures located in the region; therefore, the major goal of complete tumor resection should be achieved with a minimal rate of complications [2, 5]. Adequate preoperative work-up favors perioperative planning and improves postoperative results. Both the clinical behavior and radiological aspects of PPS tumors should be taken into consideration [8].

The aim of our study was to determine the relevant features acquired on preoperative evaluation of PPS tumors or related to the performed surgical approach that are predictive of the most important complication of the surgical treatment of these tumors, cranial nerve palsy.

## Materials and methods

The study was approved by the local Ethical Review Board. All procedures performed in the study were in accordance with the 1964 Helsinki declaration and its later amendments or comparable ethical standards.

This retrospective study included 68 patients with PPS tumors who were treated with surgical resection in a tertiary referral center from 2009 to 2019. We excluded from the analysis patients with surgical open biopsy of the PPS lesions, tumors of the deep parotid gland lobe, lateral brachial cleft cysts penetrating high into the PPS, abscesses of the PPS, pathologies originating from skull base structures, e.g., the jugular foramen, and descending into the PPS, and patients with PPS tumors revealing preoperative cranial nerve dysfunction.

The preoperative clinical signs, symptoms, age, sex, tumor size, location, histopathological type, surgical approach, radical resection, intraoperative bleeding and the occurrence of complications were collected from clinical records and follow-up examinations and then evaluated and compared. For the determination of relevant factors that may predict the most important complication of the surgical treatment of PPS tumors, deficits of the cranial nerves, we specified the variables and grouped them according to allocation as host-, tumor- or surgery-related. Age, sex, body side and reported symptoms were identified as host-related. Tumor-related features considered for analysis were localization, size, and histopathological type. Each patient was examined preoperatively with enhanced computed tomography (CT) and/or magnetic resonance imaging (MRI). The group of patients from the first analyzed years had only CT examination results, but the majority of participants had both CT and MRI information. Radiological examinations were analyzed to establish the pre- or poststyloid localization and the size of the PPS tumor. None of the patients had a preoperative cytological diagnosis of fine-needle aspiration cytology (FNAC). The final diagnosis was determined with histopathological examination of the resected tumor tissue. All enrolled patients underwent surgical treatment. Each surgical protocol was evaluated for the following surgery-related aspects: surgical approach (transparotid–transcervical, transcervical, transoral), type of surgical resection (complete, intracapsular, not radical) and severity of intraoperative bleeding (unimportant, severe). In the transparotid–transcervical approach, exposure of the facial nerve trunk and two main branches was mandatory, although parotid gland resection was optional, and sometimes only elevation of parotid tissue was described. The transcervical approach was performed either with or without submandibular gland resection and with dissection or mobilization of the posterior belly of the digastric muscle and stylohyoid muscle. Intracapsular resection was performed with incision of the tumor capsule and diminishing of tumor tissue with a dissecting instrument. Intraoperative bleeding was classified as severe when it resulted in ligation of the external carotid artery or its branches. Permanent deficits were identified when cranial nerve dysfunction was present 6 months after surgery despite rehabilitation.

Parameters were evaluated using SPSS 18.0 and Statistica 13. For all analyses, a *p* value was calculated, and values lower than 0.05 were considered statistically significant. The nominal data were evaluated in terms of independence using chi-square tests. If there were not enough observations in the contingency tables, Yates’s chi-squared tests were used.

Comparisons between numerical and nominal variables were performed with two-sample *t* tests. The normality of the distributions of the variables was confirmed using Shapiro–Wilk test.

The next step was the comprehensive analysis of factors that influenced the probability of occurrence of cranial nerve complications using logistic regression with stepwise forward selection of variables.

## Results

### Host-related factors

Among the 68 enrolled patients with PPS tumors, there were 46 (67.6%) women and 22 (32.4%) men with a mean age of 52.2 years (age range 33–92 years). Forty-one (60.3%) tumors were localized in the left PPS. Symptoms on admission were present in 43 (63.2%) patients. Most of them had foreign body sensations in the throat (35/68), difficulty swallowing (33/68), a neck mass (29/68) or symptoms of Eustachian tube dysfunction (15/68). Thirty-two percent of patients (22/68) were asymptomatic and were diagnosed accidentally, usually during posttraumatic head CT or neck, spine or head MRI (Table 1).

**Table 1:** Data of analyzed factors related to host, tumor and surgery in parapharyngeal space tumors with incidence of permanent cranial nerve deficits in the group

All patients	68
Host related factors	
Age: range, mean (years)	33–92; 52.2
Sex: women (%)/men (%)	46 (67.6%)/22 (32.4%)
Body side: left/right PPS	41 (60.3%)/27 (39.7%)
Symptomatic/asymptomatic	43 (63.2%)/22 (32.4%)
Tumor-related factors	
Localization	
Prestyloid	39 (57.4%)
Poststyloid	11 (16.2%)
Both	11 (16.2%)
Not specified	7 (10.3%)
Size	
< 4 cm/ > 4 cm	17 (25%)/51 (75%)
< 5 cm/ > 5 cm	45 (662%)/23 (33.8%)
Histopathological type	
Malignant/benign	11 (16.2%)/57 (83.8%)
Neurogenic/other	11 (16.2%)/57 (83.8%)
Types of benign diagnosis (paragangliomas and neuromas were identified also as neurogenic origin PPS tumors)	37—pleomorphic adenomas 5—paragangliomas (4 vagal nerve paragangliomas) 6—neuromas (5 vagal nerve neuromas) 9—others: lipoma, lymphangiomas, heamangiopericytoma, fibromatosis, amyloidosis
Types of malignant diagnosis	2—carcinoma ex pleomorphic adenoma 2—adenoid cystic carcinoma 2—squamous cell carcinoma 2—myoepithelial carcinomas 1—metastasis of thyroid medullary cancer
Surgery related factors	
Surgical approach	
Transparotid–transcervical	44 (64.7%)
Transcervical	21 (30.9%)
Transoral	3 (4.4%)
Type of resection	
Radical resection	59 (86.8%)
Intracapsular resection	7 (10.3%)
Non radical	1 (1.5%)
Intraoperative bleeding	
Unimportant/sever	62 (91.2%)/6 (8.8%)
Permanent cranial nerve deficits	
Vagal nerve palsy	12
Hypoglossal nerve palsy	3
Facial nerve palsy	4
Length of hospitalization	
Median; range (days)	7.8; 3–21

### Tumor-related factors

**Fig. 1:**
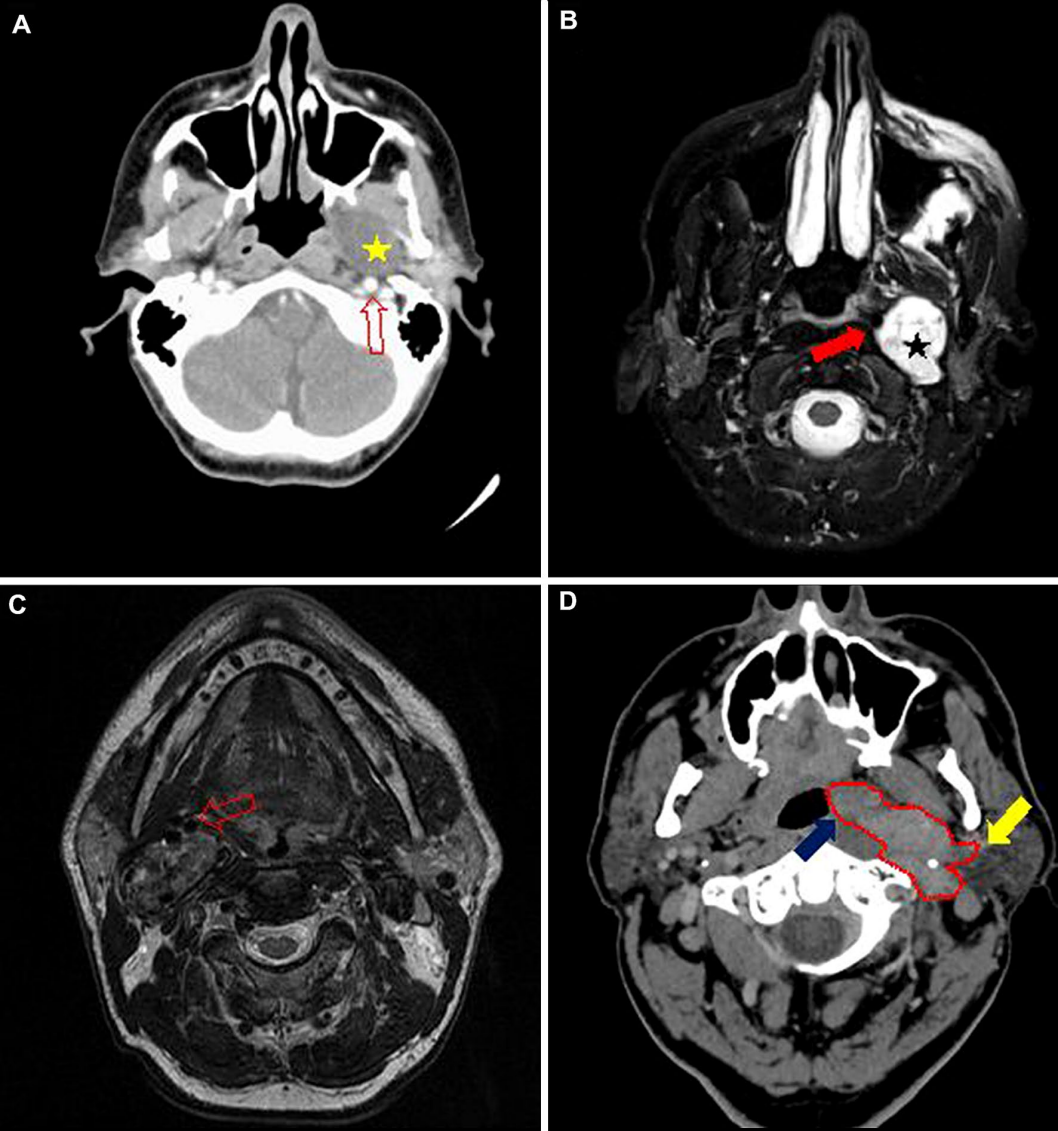
Radiological images of different parapharyngeal space tumors. **a** Axial contrast-enhanced computed tomography of a well-defined, minimally enhancing prestyloid tumor, a pleomorphic adenoma (asterisk) that posteriorly displaced the internal carotid artery (ICA) (arrow). **b** Axial T2-weighted magnetic resonance of a poststyloid space tumor, a vagal neuroma (asterisk) demonstrating homogeneous enhancement and anteromedial displacement of the ICA (arrow). **c** Axial T2-weighted magnetic resonance image of a tumor in the poststyloid compartment, a vagal paraganglioma that shows the typical “salt and pepper” sign (hypo- and hyperintense densities, relating to intratumor blood flow) and displaced the ICA anteriorly. **d** Axial contrast-enhanced computed tomography of an irregular tumor (red, dotted line) occupying both the pre- and poststyloid compartment, a squamous cell carcinoma that is weakly enhanced with contrast and presents no clear demarcation from either the lateral pharyngeal wall (blue arrow) or the parotid gland (yellow arrow)

The majority of tumors (39, 57.4%) were localized in the prestyloid portion of the PPS. There were 11 (16.2%) poststyloid tumors and the same number of tumors involving both locations. In 7 tumors, the radiological description was not sufficiently precise to determine the location, and we did not have scans for verification. Regarding the size, there were only 17 (25%) tumors smaller than 4 cm, and 23 (33.8%) were larger than 5 cm. Histology revealed 11 (16.2%) malignant tumors and 57 (83.8%) benign tumors. The categorization of origin revealed 11 (16.2%) neurogenic tumors. The specific histology of the benign tumors included 37 pleomorphic adenomas, 5 paragangliomas (4 vagal nerve paragangliomas), 6 neuromas (5 vagal nerve neuromas) and 9 cases of rare lesions, including lipomas, lymphangiomas, hemangiopericytoma, fibromatosis, and amyloidosis (Table  1, Fig. 1).

### Surgery-related factors

**Fig. 2:**
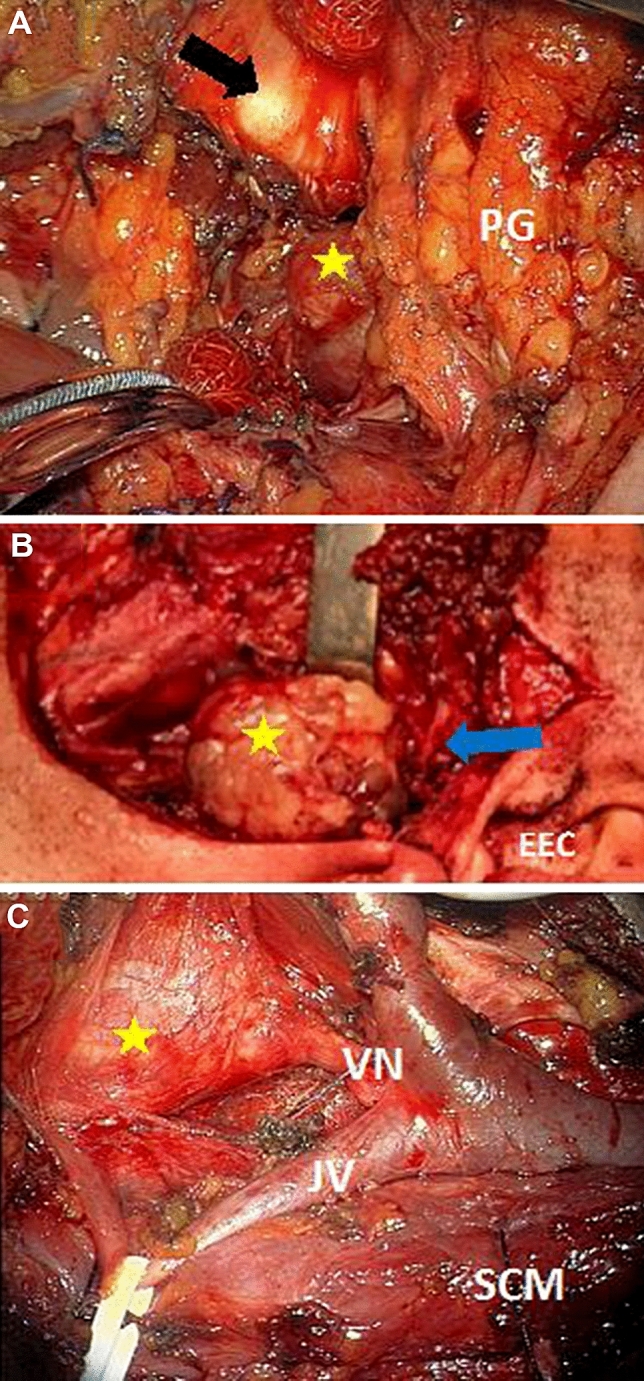
Intraoperative images of parapharyngeal space tumors resected with different approaches. **a** Transcervical approach for right-sided hemangiopericytoma (yellow arrow) resection: the submandibular gland is excised, the posterior belly of the digastric muscle is transected and the angle of the mandible is exposed (black asterisk). *PG* parotid gland. **b** Transcervical–transparotid approach for right-sided pleomorphic adenoma resection (yellow asterisk): the superficial part of parotid glad is resected, and the facial trunk and branches are identified (blue arrow). *EEC* external ear canal. **c** Transcervical approach for right-sided vagal paraganglioma (yellow arrow) resection (image from the surgical microscope): the submandibular gland is excised, the posterior belly of the digastric muscle is transected, and the lower part of the parotid gland is elevated. *VN* vagal nerve, *JV* jugular vein, *SCM* sternocleidomastoid muscle

The transparotid–transcervical approach was performed for 44 (64.7%) PPS tumors, and transcervical resection was performed for 21 (30.9%). There were only three procedures of transoral tumor removal. Complete resection of the tumor was performed in 59 (86.8%) surgeries. There were 7 (10.3%) intracapsular tumor resections. Severe bleeding occurred in 6 (8.8%) PPS tumor resections (Table 1, Fig. 2).

### Cranial nerve dysfunction

Deficits lasting longer than 6 months postoperatively were considered permanent and included vagal nerve palsy in 12 patients, facial nerve palsy in 4 patients and hypoglossal nerve palsy in 3 patients (including three patients with both X and XII nerve dysfunction).

Cross-table and chi-square test results revealed the following significant relations between nominal variables:

- Cranial nerve deficits were more common in neurogenic tumors than in other types of tumors, including malignant tumors (*χ*^2^ = 6.118, *p* = 0.013)
- The cervical approach was selected more often for neurogenic tumors (*χ*^2^ = 14.134, *p* < 0.001)
- Neurogenic tumors were more frequently removed intracapsularly (*χ*^2^ = 6.424, *p* = 0.011)
- Neurogenic tumors were more likely to be located in the poststyloid area than other types of tumors (*χ*^2^ = 17.464, *p* < 0.001) (Table 2).

**Table 2:** The cross tables and chi-square test results of relations between different factors related to host, tumor and surgery in parapharyngeal space tumors and the incidence of cranial nerve deficits (presented are results for selected features, including all significant relations)

Analyzed factors	Host related	Tumor-related					Surgery related		Final point
	Present symptoms	Malignant type	Neurogenic type	Size > 4 cm	Size > 5 cm	Poststyloid localization	Cervical approach	Intracapsular resection	Cranial nerve deficit
Present symptoms	–	0.844	0.497	0.68	0.7	0.314	0.197	0.241	0.41
Malignant type	0.844	–	–	0.995	0.346	0.245	0.999	0.999	0.65
Neurogenic type	0.497	–	–	0.825	0.939	< 0.001***	< 0.001***	0.011**	0.013**
Size > 4 cm	0.68	0.995	0.825	–	–	0.434	0.968	0.889	0.459
Size > 5 cm	0.7	0.346	0.939	–	–	0.170	0.695	0.999	0.318
Poststyloid localization	0.314	0.245	< 0.001***	0.434	0.170	–	0.018**	0.01*	0.157
Cervical approach	0.197	0.999	< 0.001***	0.968	0.695	0.018**	–	0.999	0.001***
Intracapsular resection	0.241	0.999	0.011**	0.889	0.999	0.01*	0.999	–	0.999
Cranial nerve deficites	0.41	0.65	0.013**	0.459	0.318	0.157	0.001***	0.999	–

*p < 0.1; **p < 0.05; ***p < 0.01

There was also a significant relationship between numeric and nominal variables. The two-sample *t* test revealed significant differences in the mean ages of patients between groups with and without cranial nerve complications (*t* = 2.242, *p* = 0.031). The assumption of a normal distribution was confirmed in both groups using Shapiro–Wilk tests (*p* values 0.183 and 0.914, respectively). The mean age in the group of patients with cranial nerve complications was 45.89 years, and that in the group without complications was 54.69 years. Older patients rarely presented with cranial nerve complications.

The occurrence of cranial nerve dysfunction was highly correlated with the type of PPS tumor. According to the results of logistic regression for the neurogenic tumors, the probability of complications was almost 8 times higher than for other tumor types (OR = 7.778, *p* = 0.01). None of the other analyzed variables related to tumor or surgery was significantly correlated with increased cranial nerve dysfunction (Table 3).

**Table 3:** The results of logistic regression model with stepwise selection of variables analyzing the influence of different factors related to host, tumor and surgery on the risk of cranial nerve complications in parapharyngeal space tumors

Analyzed variable	*p* value	OR
Significant variables		
Constant	< 0.001	0.257
Neurogenic tumor	0.010	7.778
Insignificant variables		
Sex	0.092	2.832
Age	0.153	2.037
Malignant tumor	0.143	2.141
Size > 4 cm	0.385	0.755
Size > 5 cm	0.325	0.970
Localization	0.379	0.775
Symptoms before resection	0.256	1.288
Surgical approach	0.103	2.666
Method of resection	0.638	0.222
Bleeding	0.400	0.708

## Discussion

The challenge of PPS tumor resection is to completely remove the lesion with a minimal number of postoperative complications, especially those related to neural deficits. The accurate identification of factors predisposing the patient to an unsuccessful course will enable preoperative assessment of the patient's attitude, counseling and determination of an optimal plan for treatment and recovery. Our study aimed to identify relevant features that could be acquired on preoperative evaluation of PPS tumors or that are related to the surgical approach performed. We analyzed a series of 68 patients treated for PPS tumors. The distribution of histopathological types in our group was in accordance with data from the literature on the proportion of malignant tumors 16%, compared to 18% in a review of 1118 cases by Riffat et al. [2]. However, the incidence of neurogenic lesions among benign tumors (16%) was lower than that in the mentioned study (41%). In turn, our study revealed a higher rate for pleomorphic adenoma (64% vs. 45%) [2]. In contrast, a cohort analysis by some authors indicated a greater proportion of neurogenic PPS tumors than salivary gland tumors in their populations [4, 10, 11].

The mean age of patients treated for PPS tumors ranges from 53 [12] to 42 years [10]; however, some authors include patients under 18 years of age in the analysis. Our population mean age was 52 years. Regarding factors related to the host, we found that younger patients were more likely to present with cranial nerve dysfunction postoperatively. The symptoms of PPS tumors are diverse and nonspecific. The most common signs in our study were foreign body sensations in the throat, difficulty swallowing and the presence of a neck mass, which are comparable with data from previous reports [2, 10, 12]. However, the most challenging aspect for surgical treatment is the group of patients with PPS tumors that report no symptoms, especially as it may involve between 26% [12] and 43% [10] of treated patients. Our analysis, which included 32% asymptomatic cases, did not reveal either an increased risk or preferable outcomes for the presence or absence of symptoms.

Preoperative information regarding PPS tumors is preferably obtained with radiological imaging, and a cross-sectional work-up involving both CT and MRI is currently recommended for complimentary evaluation of the tumor interface, vascularization, and relationship to tissue of the parotid gland and other surrounding structures, including the skull base. Before planning the resection, the surgeon should primarily focus on the demarcation of the tumor from the surrounding structures, the compartment of localization (pre- or poststyloid), the size and the evidence of malignancy. These features are important for selecting the approach for resection. Prestyloid tumors commonly originate from salivary tissue, while poststyloid tumors are most often derived from neurological structures. In our study, the majority of tumors (39, 57.4%) were located in the prestyloid space. There were 11 (16.2%) postyloid tumors and the same number of tumors involving both localizations. We identified that neurogenic tumors were more likely to be located in the poststyloid area than other types of tumors. The concealed location of PPS tumors and their slow growth contribute to the rather large volume at diagnosis. In our study, only 25% of the tumors were smaller than 4 cm, and tumors larger than 5 cm accounted for 33.8%. However, the hypothesis that a larger tumor size may determine postoperative neurological complications was not proven with our comparison. Most authors present only the measures for the largest tumors in their series but do not analyze the cumulative influence of tumor size on the outcomes; for example, Sun et al. found that the longest mean diameter of PPS tumors was 5.6 cm, Chang et al. reported a largest tumor size of 6.8 cm among their 51 cases, and Presutti et al. described an 8 cm diameter as the largest [10, 13, 14]. Of the factors related to the tumor, only neurogenic origin by histopathology was found to be a significant predictor of neural deficits in the postoperative period. Surprisingly, malignant PPS tumors were not associated with an increased complication rate.

The authors of previous publications suggest numerous guidelines that should be followed in the selection of a transcervical, transparotid–transcervical or transoral approach for PPS tumor surgery. According to the review of 686 surgical cases by Riffat et al., the transcervical approach is reported as the most frequently used and accounts for 48% of all approaches [2]. In our analysis, nearly 65% of PPS tumors were resected with a transparotid–transcervical approach; however, the cross-table and chi-square test results revealed that cervical access was more often selected for neurogenic tumors, which is in agreement with previous studies [11, 15, 16]. Intracapsular resection of PPS tumors, performed especially for neuromas, remains controversial. This less traumatic technique enables preservation of nerve function at the expense of nonradical resection and deviation from the oncologic principle of en bloc resection. Our comparison did not reveal significant differences between the effects of complete and intracapsular resection on postoperative nerve function; however, the sample of intracapsular cases was quite small. In the future, the widespread application of electromyographic (EMG) mapping of nerve fibers may substantially reduce damage to nerve fibers during surgical enucleation of neuromas [17–19]. The enucleation of pleomorphic adenomas is also controversial among authors due to the risk of tumor spillage and recurrence. Another factor related to surgery of PPS tumors that we included in the analysis was severe bleeding during the operation that required external carotid ligation. In our series, this factor had no significant influence on postoperative complications.

We did not analyze the accuracy of fine-needle aspiration biopsy (FNAB) in the preoperative elaboration of PPS tumors because, in our opinion, they are unusual targets for biopsy. The disadvantages of FNAB include difficulty of access, risk of vascular complications, and hemorrhagic or hypocellular specimens in neurogenic tumors. We agree that FNAC of PSS tumors should be considered for selected prestyloid lesions with radiological suspicion of malignancy [20].

In recent decades, robotic- and endoscopic-assisted transoral techniques have expanded the range of approaches for PPS tumors. Both methods involve, however, a quite high rate of tumor fragmentation (15–24%), and most studies lack data on long-term outcomes, especially regarding recurrence [21, 22].

Currently, there is much controversy regarding PPS neurogenic tumor treatment options. Primary surgical resection is frequently questioned, especially in cases of asymptomatic, slowly growing tumors, in favor of monitoring and observing approaches. The supporters of conservative treatment claim that a gradual impairment of cranial nerve function is better tolerated than sudden paralysis from surgery, especially for elderly patients [23, 24]. The final decision should depend on the patient’s preference after informed consent. Evidently, pre-existing contralateral dysfunction of the vagal or hypoglossal nerve in patients with benign poststyloid PPS tumors should be considered an indication for an optional treatment method aside from surgery due to the high risk of bilateral cranial nerve paresis and swallowing and airway problems demanding permanent tracheostomy and tube feeding. In those cases, radiotherapy may be considered a primary treatment option. Other indications for radiotherapy include postoperative cases of malignant, aggressive histology or inadequate margins.

The potential sequelae of postsurgical cranial nerve deficits following the resection of PPS tumors require the consideration of concurrent or subsequent reconstructive methods involving reinnervation, vocal fold augmentation or medialization thyroplasty for vagal nerve deficits and dynamic or static techniques of facial animation for facial nerve deficits.

Another recognized and rare complication of parapharyngeal space surgery of still unrevealed mechanism, yet with a hypothetically possible neurological basis, is first bite syndrome. The most likely theory of its origin is the destruction of the cervical sympathetic plexus along the carotid artery and the sympathetic denervation of the parotid gland during the surgical resection of PPS tumors [25]. In our study, only 3 patients (two with pleomorphic adenoma, one with vagal paraganglioma) presented the typical sharp pain with the first bite of the meal. The severity of pain deceased spontaneously with time, and in all cases, the symptoms subsided within 6 months post operation without treatment.

The retrospective design of our study limits the overall significance of the results. Therefore, because of the rarity of PPS tumors, multicenter prospective studies involving preoperative assessment with imaging methods and surgical approaches, including endoscopic and robotic techniques as well as nerve monitoring and therapeutic options for restoration of involved nerve function, need to be planned for developing optimal therapeutic algorithms.

## Conclusion

The study revealed that the relevant factors for developing postoperative sequelae of cranial nerve paralysis are related to tumor neurogenic origin and younger patient age.

Therefore, efforts should be concentrated on the preoperative radiological identification of PPS tumors in the poststyloid compartment and comprehensive information and counseling for these patients about potential outcomes involving neurological complications and possible methods of postoperative speech and swallowing therapy in case of lower cranial nerve paralysis.

The surgical resection of tumors other than neurogenic tumors of PPS revealed no significant risk for permanent neural dysfunction. Tumor size also had no significant effects on the risk of postoperative nerve palsy.

## Data Availability

On request.
